# Quaternized cellulose nanofibril paper coatings for sustainable packaging with improved barrier and antibacterial performance

**DOI:** 10.1038/s41598-026-48158-2

**Published:** 2026-04-09

**Authors:** Yoon-hyuck Choi, Seungmin Han, Soo-jeong Shin, Zhenghui Shen, Araz Rajabi-Abhari, Jaeho Ryu, Wanhee Im

**Affiliations:** 1 Department of Wood & Paper Science, Chungbuk National University, Chungdae-ro 1, Seowon-gu, Chengju, 28644 South Korea; 2 State Key Laboratory of Advanced Waterproof Materials, School of Materials Science and Engineering, Peking University, Beijing, 100871 China; 3 Department of Chemical & Biological Engineering, University of British Columbia, 2360 East Mall, Vancouver, BC V6T 1Z3 Canada; 4 Research and Development Center, Greeneple Inc, 21 Kentech-ro, Naju, 58330 South Korea

**Keywords:** Cellulose nanofibrils, Cationic group contents, Rheological behavior, Quaternization, Air barrier, Antibacterial performances, Materials science, Microbiology, Nanoscience and technology

## Abstract

Quaternized cellulose nanofibrils (quaternized CNFs) with different cationic group contents were prepared and applied as multilayer paper coatings to develop sustainable antibacterial and barrier materials. Increasing the cationic group content (160–660 µmol/g) reduced CED viscosity and yield stress, indicating weakened network structures and reduced interfibrillar interactions These rheological changes improved coating flow and layer uniformity, leading to denser coating layers with enhanced air barrier performance and water resistance. Antibacterial tests revealed clear contact-active antibacterial activity against Gram-positive bacteria, which increased with cationic group content and coating layer number, likely because the immobilized quaternary ammonium groups more effectively interacted with the exposed negatively charged cell membrane. In contrast, antibacterial performance against Gram-negative bacteria remained limited, likely due to the protective outer membrane. Within the investigated range, a maximum cationic group content of approximately 660 µmol/g was sufficient for Gram-positive bacteria, whereas higher contents may be required for effective activity against Gram-negative bacteria.

## Introduction

Growing concerns over plastic pollution and the depletion of non-renewable resources have intensified interest in bio-based and biodegradable materials. There is a strong need to develop functional biomaterials that ensure both human safety and environmental compatibility across various fields, including food packaging, hygiene products, and biomedical applications. Among the diverse candidates, cellulose-based nanomaterials have attracted significant attention due to their unique combination of abundant availability, biodegradability, recyclability, high mechanical strength, and ability to be chemically modified for functional applications^1,2^.

Cellulose nanofibrils (CNFs) have been actively explored as paper coating materials owing to their excellent film-forming ability and compatibility with water-based systems, and capacity to form dense fibrillar networks that enhance barrier performance^3^, which plays a vital role in protecting packaged products. Their excellent film-forming ability contributes to reduced air permeability and enhanced surface smoothness, leading to improved printability and increased resistance to grease and water vapor^4,5^. Furthermore, the nanofibrillar layers formed by CNFs contributes to surface mechanical reinforcement, which can enhance the overall durability of the coated paper^6,7^. These properties make CNFs promising alternatives to petroleum-based coatings in paper packaging.

In the field of sustainable packaging, there is a growing demand for antibacterial coating materials as a means to prevent microbial contamination and extend the shelf life of perishable products^8,9^. However, conventional antibacterial agents, including silver nanoparticles, zinc-based compounds, and organic biocides, are limited by leaching behavior, which may result in cytotoxicity, environmental accumulation, and reduced long-term efficacy^10–12^. Recent studies on biomass-based active packaging have demonstrated that multifunctional biodegradable films can be developed by integrating antimicrobial and preservation-related functions into renewable polymer systems. For example, biomass-based active films have been reported to simultaneously provide non-leaching antibacterial activity, biodegradability, antioxidant performance, and food-preservation capability through all-natural polymer systems or multifunctional bio-based film designs^13,14^. Nevertheless, these studies have mainly focused on the overall functionality of active films, whereas relatively little attention has been paid to how non-releasing nanofibrillar coating materials, particularly quaternized CNFs, influence coating-layer formation and functional performance in paper packaging.

In this regard, quaternary ammonium compounds have emerged as promising candidates because they can provide contact-active (non-releasing) antibacterial effects through immobilized quaternary ammonium cationic groups^15–17^. The antibacterial mechanism of these cationic compounds is primarily attributed to their contact-killing effect. Namely, cationic compounds are initially adsorbed onto negatively charged microbial cell surfaces through electrostatic interactions, followed by the detachment of anionic phospholipids and destabilization of the cytoplasmic membrane^18,19^. This leads to uncontrolled leakage of intracellular components and ultimately causes cell death^20,21^. Therefore, introduction of quaternary ammonium groups offers a promising strategy for developing sustainable active packaging materials with durable antibacterial functionality while reducing potential environmental and health hazards^10^.

Among the chemical pretreatments for preparing CNFs, the quaternization reaction introduces cationic quaternary ammonium groups on the cellulose surface^22–26^. This cationic functionalization not only facilitates nanofibrillation by increasing electrostatic repulsion between cationic fibrils^23,27,28^, but also provides additional functionalities, including antimicrobial activity and adsorption of anionic substances^25,27,29,30^. Our previous studies demonstrated that the cationic group content of quaternized CNFs could be tuned by changing the quaternization conditions^31,32^. Although quaternized CNFs have been extensively studied, the role of cationic group content in controlling fibril morphology, rheological behavior, coating layer formation, and the resulting antibacterial and barrier performance has not yet been fully clarified.

Therefore, in this study, we aimed to investigate the effects of cationic group content, controlled by the dosage of glycidyltrimethylammonium chloride (GMA), on the morphology and rheological behavior of quaternized CNFs as coating materials for sustainable paper packaging. In addition, the influence of cationic group content on coating layer formation, barrier properties, and contact-active antibacterial performance of CNF-coated papers was investigated. These findings are expected to provide useful insights into the design of sustainable and practically relevant active coating materials based on renewable cellulose resources and water-based coating systems.

## Materials and methods

### Materials

To prepare the quaternized CNFs with different cationic group contents, never-dried bleached eucalyptus kraft pulp supplied by Moorim P&P (Korea) was used as the starting material. The pulp was thoroughly washed with deionized water and mechanically disintegrated using a stirrer operating at 1000 rpm. The resulting slurry was then concentrated to a consistency of 31 wt% using vacuum filtration prior to quaternization. The chemical composition of the pulp fibrils was determined according to NREL test method (TP-510–42618), revealing contents of 79.5 ± 0.5% cellulose, 18.5 ± 0.4% hemicellulose, and trace amounts of lignin and ash. For the quaternization reaction, sodium hydroxide (NaOH, CAS: 1310-73-2, Mw = 40.00, Samchun Chemical, 98.0%) and glycidyltrimethylammonium chloride (GMA, CAS: 3033-77-0, Mw = 151.63, Sigma-Aldrich, ≥ 90%) were employed as reaction reagents and isopropanol (IPA, CAS: 67-63-0, Mw = 60.10, Duksan Reagents, 99.5%) was used as the solvent medium. In addition, silver nitrate (AgNO₃, CAS: 7761-88-8, Mw = 169.87, Sigma-Aldrich, ≥ 99.0%) was used as titration to determine the cationic group contents of quaternized CNFs. For the antibacterial assays, bacterial strains were obtained from the Korean Collection for Type Cultures (KCTC). The strains included *Escherichia coli* (Gram-negative, KCTC 1023), *Bacillus subtilis* (Gram-positive, KCTC 1481), and *Staphylococcus aureus* (Gram-positive, KCTC 1621).

## Methods

### Quaternization of pulp

Quaternization of pulp was carried out according to previous research^32^. Briefly, 5 g of pulp fiber (31 wt% consistency) was placed in a polyethylene bag along with a mixed solvent of IPA and deionized water (3:1, v/v), followed by the addition of NaOH at 10 wt% based on oven-dried weight of the pulp. After moderate mixing for 10 min, GMA was added to the pulp paste at the respective concentrations of 8, 10, 12, 14, and 16 mmol/g, which correspond to the preparation of quaternized pulps, i.e. QCNF_1, QCNF_2, QCNF_3, QCNF_4, and QCNF_5, respectively. The reaction was conducted at 45 °C for 1 h under continuous stirring. After completion, the pretreated pulp fibers were thoroughly washed with deionized water until the pH reached 7.0 ± 0.5 and the conductivity was below 20 µS/cm.

**Fig. 1:**
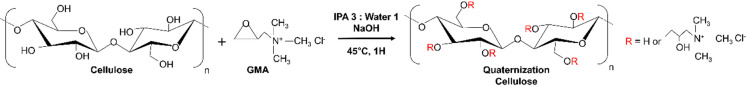
Reaction scheme of GMA-based quaternization of cellulose.

## Preparation and characterizations of quaternized CNFs

The pre-treated pulps obtained under different reaction conditions were diluted with deionized water to a consistency of 0.8 wt% and a total volume of 0.5 L to produce quaternized CNFs. The pulp suspension was then processed using a high-pressure homogenizer (GEA, Niro Soavi Panda Plus, Italy) at 900 ± 50 bar for 5 passes to achieve sufficient nanofibrillation.

Transmission electron microscope (TEM, Carl Zeiss, LIBRA 120, Germany) was used to evaluate the morphological properties of quaternized CNFs. For TEM analysis, CNFs suspension was diluted with deionized water to 0.001 wt% and the deposited on a glow-discharged Cu arid (Carbon type-B, Ted Pella Inc.), followed by negative staining using uranyl acetate. The average width of quaternized CNFs prepared by different reaction conditions was evaluated using ImageJ software. Moreover, the cupriethylenediamine (CED) viscosity of the quaternized CNFs was measured to investigate the degree of polymerization according to the TAPPI standard (T230om-08).

The cationic group content of quaternized CNFs were determined by conductometric titration using AgNO_3_ following the method described by Hasani et al.^26^. For the titration, 0.05 g of dried quaternized CNF was dispersed in 100 mL of deionized water under continuous stirring and titrated with 5 mM AgNO₃ solution added dropwise at 30-second intervals. Conductivity was continuously monitored during the titration, and the endpoint was identified when conductivity began to increase linearly. The cationic group contents were calculated using Eq. (1):

Cationic group content = (V × C_AgNO3_)) /m (1).

where V is the volume of AgNO_3_ solution added (mL), CAgNO_3_ is the concentration of AgNO_3_ solution (mol/L), and m is the mass of dried quaternized CNFs (g).

The rheological properties of quaternized CNF were analyzed using a rheometer (MCR 102, Anton Paar, Austria) equipped with a parallel plate geometry (D-PP25) and a 1 mm plate gap. Rotational viscosity as a function of shear rate was measured using a 0.6% suspension of quaternized CNFs. The oscillatory mode of the rheometer was used to investigate the network properties of quaternized CNFs suspension. A shear strain amplitude sweep was performed in the range of 0.01–100% strain at a constant frequency of 10 rad/s.

## Fabrication of quaternized CNFs coated paper

To investigate the effect of cationic group contents on the properties of quaternized CNFs coated papers, quaternized CNF were diluted to a consistency of 0.6 wt% using deionized water and coated onto base paper with a grammage of 280 g/m² using a rod coater (GBC-A4, Gist Co., Korea) and rod bar number 47. The CNFs coating was applied in two or four layers, with each layer followed by drying at 105 °C.

### Characterizations of quaternized CNFs coated paper

Field emission scanning electron microscopy (FE-SEM, Carl Zeiss Gemini 560, Germany) was used to examine the surface morphology of paper coated with quaternized CNFs. For FE-SEM analysis, coated paper samples were cut into 10 × 10 mm pieces, mounted on aluminum stubs using conductive carbon tape, and sputter-coated with platinum to a nominal thickness of 5 nm. Imaging was performed under high vacuum at an accelerating voltage of 3 kV and a working distance of 4.5 mm, and micrographs were captured at multiple magnifications to assess coating uniformity.

Prior to air permeability and contact angle measurements, all specimens were conditioned at 23 ± 1 °C and 50 ± 2% relative humidity for at least 24 h, and the tests were conducted under the same environmental conditions.

The air permeability of coated and uncoated samples was measured using an air permeability tester in accordance with TAPPI T 460 om-96.

The surface wettability of quaternized CNFs coated papers was evaluated using a contact angle goniometer (Phoenix-300 Touch, Surface Electro Optics, Korea). A 5 µL droplet of deionized water was carefully placed onto the sample surface, and the static contact angle was measured at time intervals from 0 to 150 s, with measurements recorded every 30 s.

Antibacterial activity was evaluated using both the colony forming unit (CFU) method^33^ and shaking flask method^19^. Quantitative antibacterial activity was assessed by the colony forming unit (CFU) counting method. 100 µL of the bacterial suspension was spread onto an agar plate, and the paper sample was placed with the coated surface facing the agar. After incubation at 37 °C for 18–24 h, the agar under the coated paper was excised and transferred into 10 mL of distilled water. The suspension was then mixed thoroughly to extract the bacteria, serially diluted to 10⁻⁶, and 100 µL of each dilution was plated onto agar. After additional incubation at 37 °C for 18 h, colonies were counted and CFU values were calculated using the following Eq. (2):

CFU/mL = (C×D)/V (2).

where C is the number of colonies, D is the dilution factor, and V is the plated volume (mL).

For the shaking flask test, bacterial suspensions were diluted 1:100, followed by a serial dilution to 10⁻⁶. The quaternized CNFs coated paper samples were cut into 10 mm × 30 mm strips and immersed in the diluted bacterial suspensions. After that, coated paper samples were additionally incubated at 37 °C in a shaking water bath for 1 h. After incubation, 100 µL of the bacterial suspension was plated onto agar and incubated at 37 °C for 18–24 h to qualitatively assess antibacterial activity, and representative images were subsequently captured.

## Results and discussion

### Effect of cationization on quaternized CNF properties

**Fig. 2:**
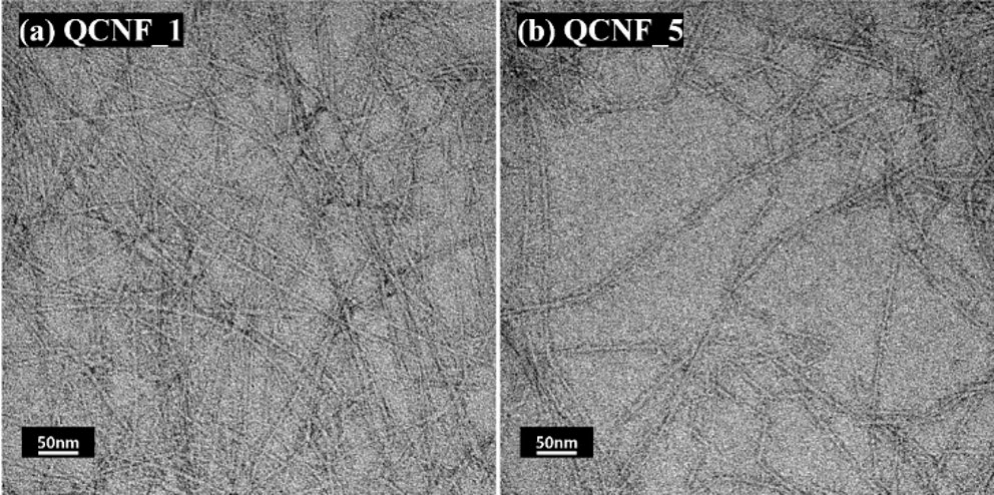
TEM images of quaternized CNFs with different cationic group contents.

To investigate the effect of cationic group content on the characteristics of CNFs, the GMA addition level was systematically controlled. Table 1 summarizes the cationic group content, CED viscosity, and fibril width of quaternized CNFs prepared at different GMA addition levels. The cationic group content of each sample was quantified by AgNO₃ conductometric titration and was found to increase from 160 µmol g⁻¹ to 660 µmol g⁻¹ as the GMA dosage increased from 8 to 16 mmol g⁻¹ (based on dry pulp). As summarized in Table 1, the CED viscosity decreased with increasing cationic group contents. Because CED viscosity is generally used as an indicator of the degree of polymerization (DP) of cellulose, this result suggests that cellulose depolymerization occurred to a greater extent under higher GMA treatment conditions^34^. Several previous studies shown that decreases in DP are associated with shortened CNF length after chemical pretreatment. Therefore, the reduced CNF length is more appropriately attributed to reaction-induced degradation of cellulose during the cationization process, rather than to the cationic substitution itself^28,32,35^ In contrast of DP, the fibrils width of quaternized CNFs had similar width at approximately 8.7 nm, regardless of cationic group contents (Table 1; Fig. 2). This indicates that even at a cationic group contents approximately 160 µmol/g is sufficient to induce electrostatic repulsion that allows effective nano-fibrillation after five HPH passes.

**Table 1:** Characterization of quaternized CNFs with different cationic group contents.

Classification	Cationic group content, µmol/g	CED Viscosity, mPa‧s	DPw	Width, nm	
QCNF_1	160 ± 12	5.11 ± 0.34	911.53	8.4	
QCNF_2	260 ± 10	4.82 ± 0.12	862.78	9.0	
QCNF_3	380 ± 15	4.48 ± 0.15	801.74	8.7	
QCNF_4	620 ± 25	4.15 ± 0.18	737.89	8.5	
QCNF_5	660 ± 20	4.11 ± 0.15	729.81	8.5	

Rheological measurements were conducted to assess the effect of the estimated reduction in fibril length, resulting from increased cationic group contents, on the flow behavior of quaternized CNFs suspensions.

**Fig. 3:**
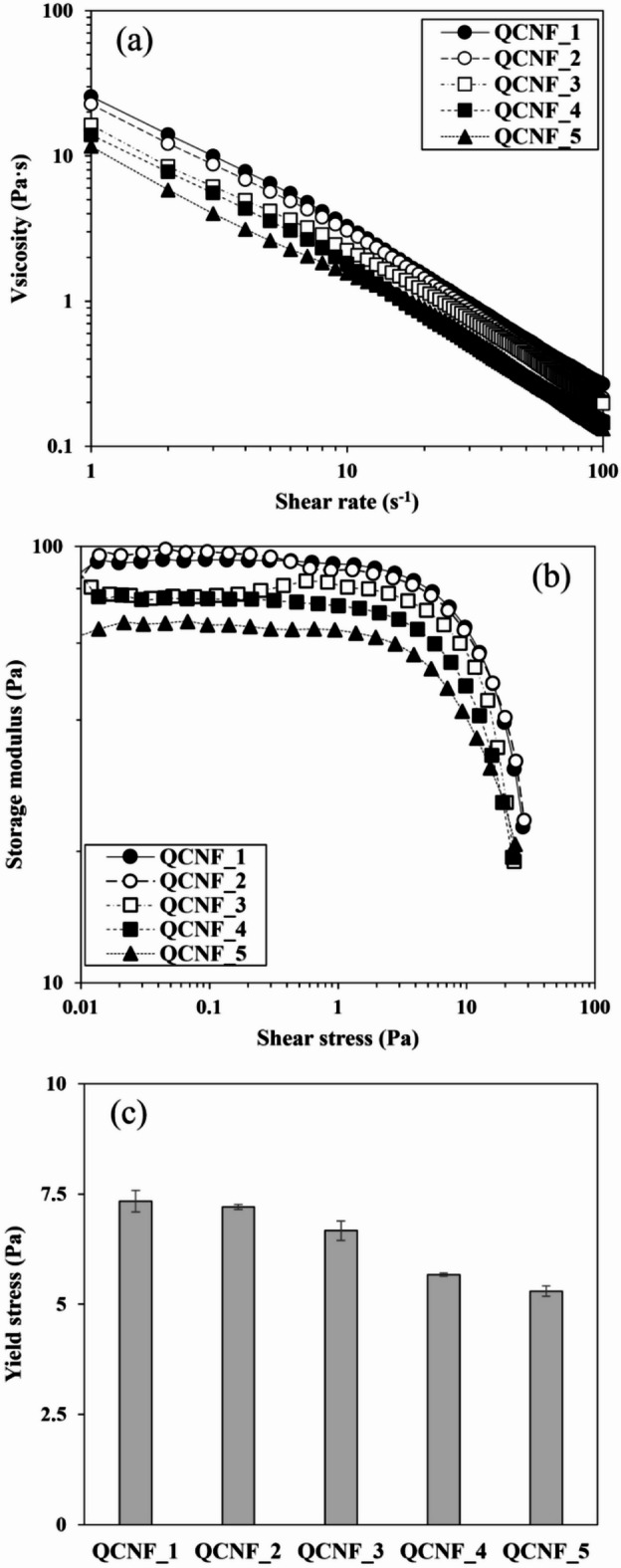
Rheological properties of QCNFs with different cationic group contents. Viscosity as a function of shear rate (**a**), Storage modulus (G′) as a function of shear stress (**b**) and yield stress values (**c**).

Figure 3a presents the shear viscosity of the quaternized CNFs samples. Shear thinning behavior was clearly observed, which was caused by the interactions between fibrils orientated along the shear direction. In addition, shear viscosity decreased with increasing cationic group contents. This trend can be attributed to the reduced fibril length inferred from the CED viscosity in Table 1, as shorter fibrils restrict entanglement and network formation in CNF suspension^36^.

The amplitude sweep mode measurement was also performed to investigate the network strengths of quaternized CNFs prepared with different cationic group contents, Fig. 3b plots the storage modulus of CNFs against shear stress. The storage modulus had a plateau region in the low shear stress range. It can be seen that the storage modulus in the plateau region decreases as the cationic group contents of CNFs increase. Generally, a lower storage modulus indicates that the materials have a weaker elastic network structure and reduced ability to store deformation energy under stress. Thus, these results indicated that the fibrillar network is less interconnected between the fibrils due to the reduction in length, which may result in decreased mechanical stability and structural integrity^37–40^. Furthermore, the storage modulus exhibits a significant decrease in the shear stress region above a critical level as shown in Fig. 3c. The shear stress at which the storage modulus drops sharply is commonly defined as the yield stress, an indicator of network strength that is associated with the beginning of network breakdown once this threshold is reached^36^. As shown in the results, an increase in introduced cationic group contents leads to a decrease in the yield stress of the CNFs suspension. This rheological analysis clearly indicates that an increase in cationic group contents leads to a reduction in CNFs length.

### Air barrier and water-resistance of quaternized CNF-coated paper

The smoothness and uniformity of surface onto coated paper are key factors that influence air barrier and water-resistance properties^41^. Therefore, to investigate the effect of CNFs coatings with different cationic group contents on coated paper, quaternized CNFs were applied onto the base paper in two and four coating layers.

**Fig. 4:**
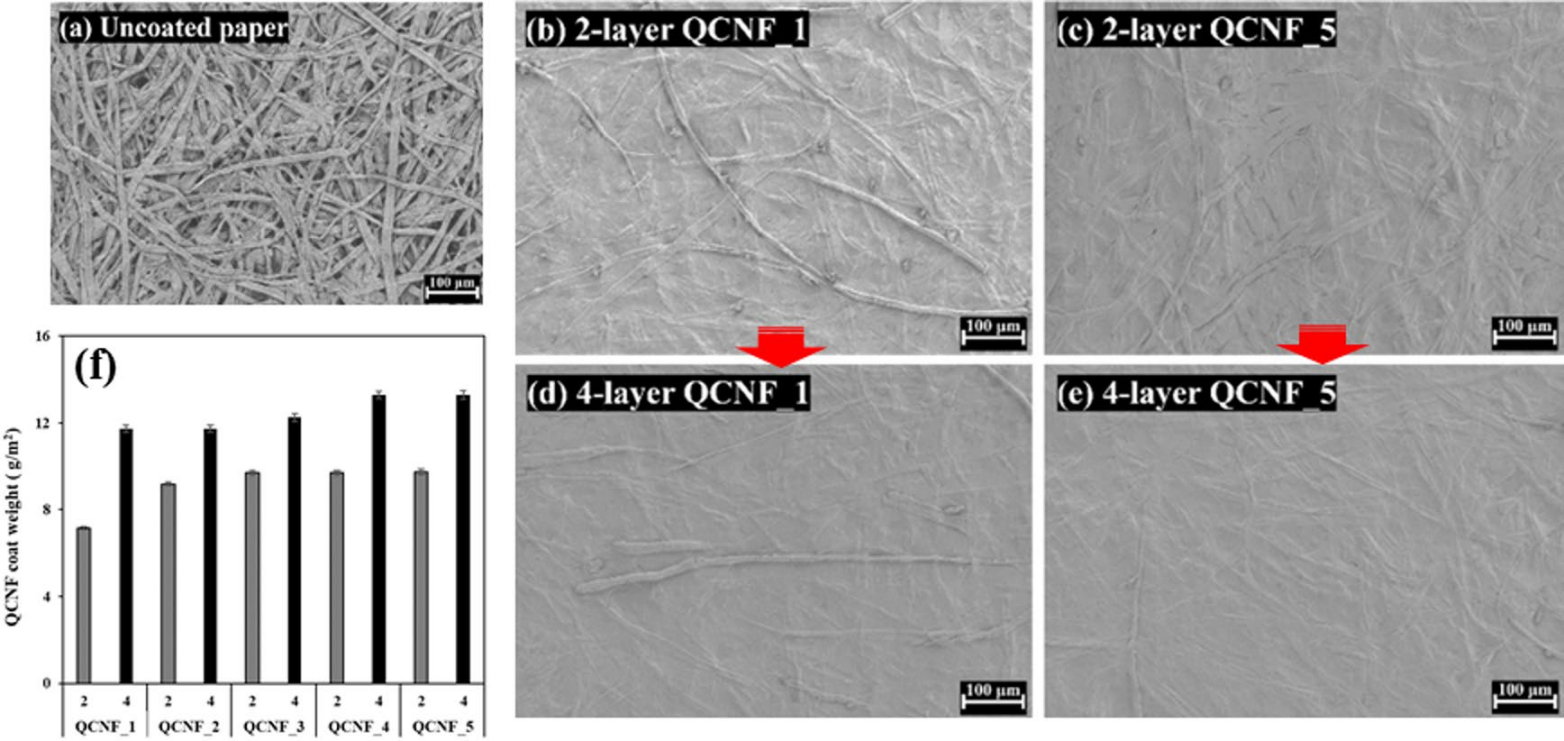
FE-SEM image of QCNF coated paper surfaces and coated weight of CNFs depending on cationic group contents.

Figure 4a-e show FE-SEM images of the surface of the base paper and papers coated with QCNF_1 (lowest cationic group content) and QCNF_5 (highest cationic group content) with two and four coating layers, respectively. As shown clearly, the surface uniformity of the coated paper improved with increased coating layer. In addition, it is noteworthy that, the surface uniformity of CNFs coated paper differed significantly depending on cationic group contents of CNFs, even at the same coating number. In the case of coated paper four times with QCNF_1, surface irregularities of base paper remain partially observed. In contrast, for QCNF_5, which had the highest cationic group contents, most of the surface of base paper is effectively covered by the quaternized CNFs coating layer.

Figure 4f presents the coating weight of CNFs applied onto the base paper using the same rod. As shown in the figure, the coating weight gradually increases with increasing cationic group contents of quaternized CNFs. This trend can be attributed to the improved flow behavior of CNFs suspension evaluated rheological results (Fig. 3). That is, the increased cationic group content improves the flowability of CNF suspensions through a reduction in fibril length, which consequently enables a higher coating amount to be applied under identical coating conditions^42,43^.

**Fig. 5:**
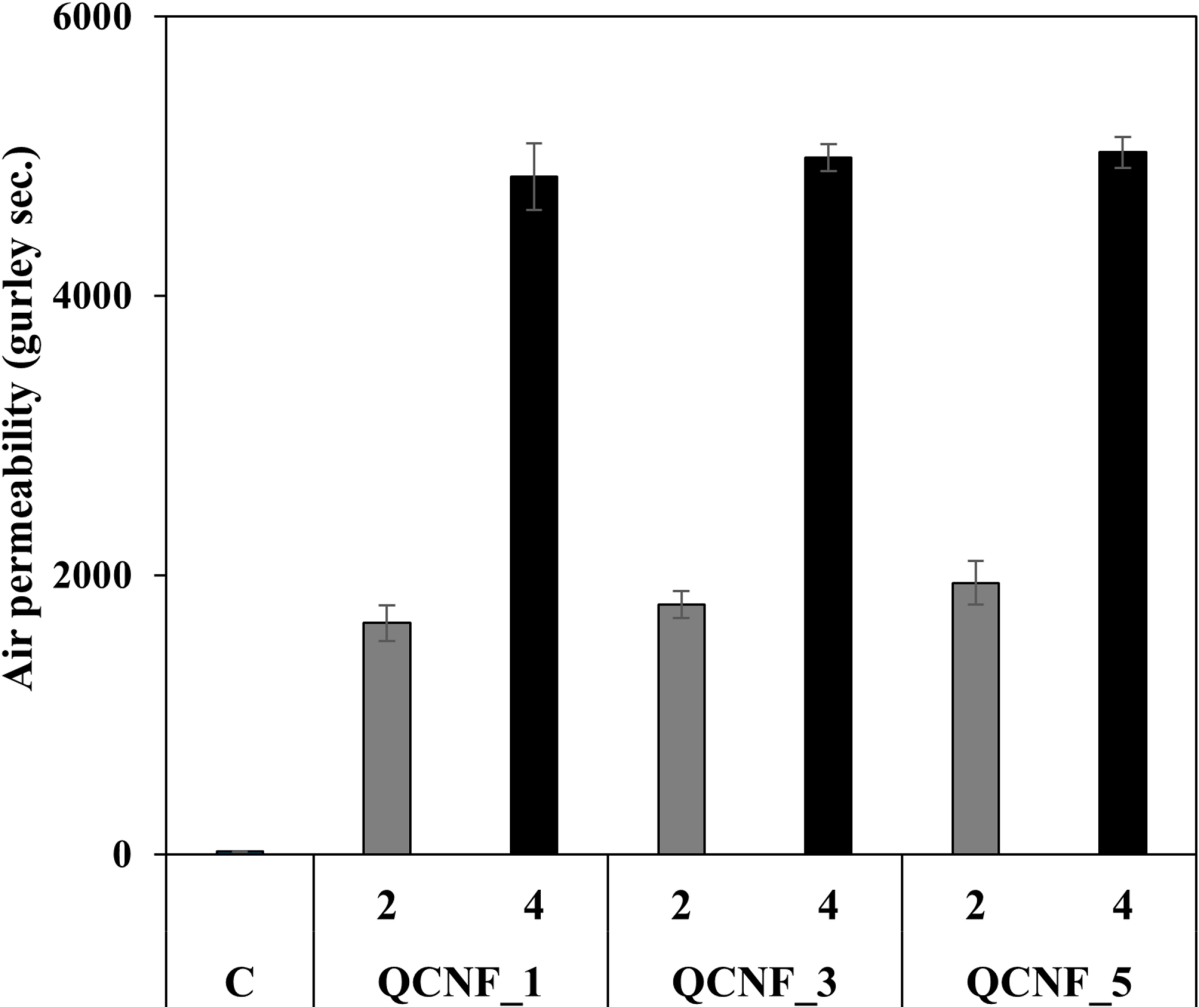
Air permeability of coated paper depending on QCNFs content and coating layers. C presents uncoated paper.

Figure 5 shows the air permeability of papers coated with quaternized CNFs. Both the two layer and four-layer coatings exhibited improved air barrier properties compared to the uncoated paper. In case of coated paper with two layers, the air barrier performance tended to improve with increasing cationic group contents due to the improved surface coverage. However, for the four-layer coated samples, the air barrier properties were nearly same regardless of CNFs preparation conditions, which is likely due to the sufficient surface coverage. Previous studies by Afra et al.^44^ and Aulin et al.^45^ also demonstrated that multilayer CNFs coatings can more effectively block surface pores and form a dense, uniform barrier, resulting in significant improvement in air barrier performance.

**Fig. 6:**
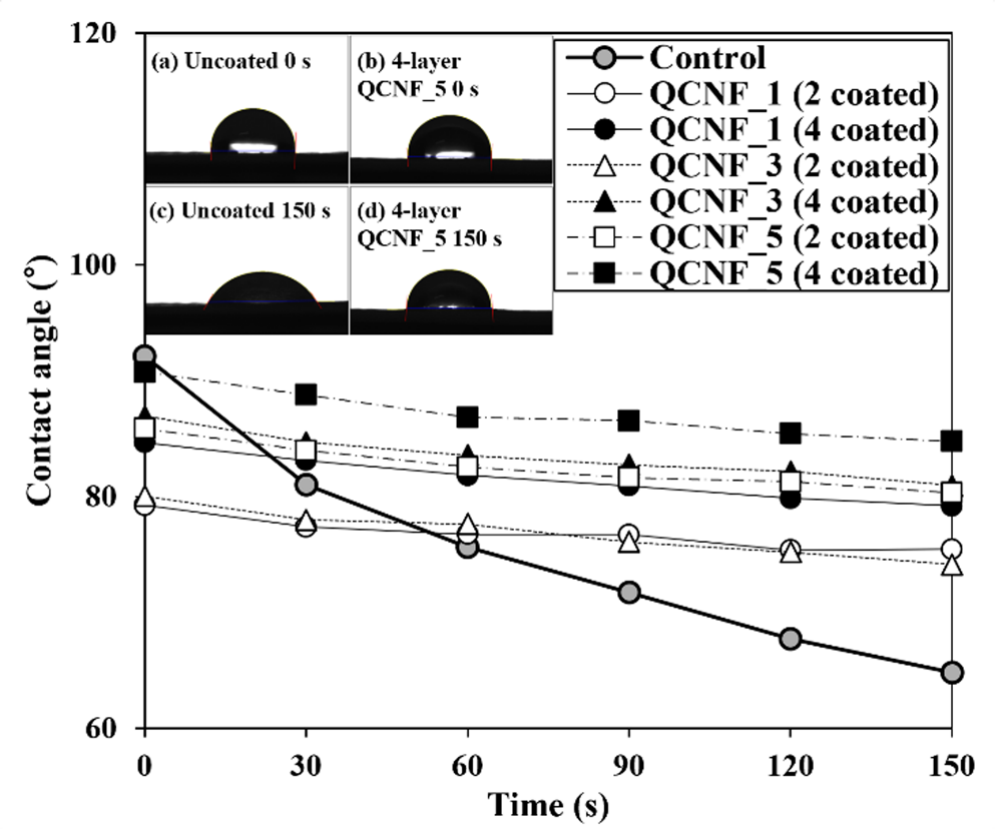
Time-dependent contact angle of quaternized CNFs-coated papers.

Figure 6 shows the time-dependent contact angles of papers coated with quaternized CNFs. The contact angle was measured every 30 s for 150 s to evaluate water resistance. First, the papers coated with quaternized CNFs exhibits higher water resistance as a function of time compare to base paper (Uncoated paper). In addition, papers coated with quaternized CNFs maintained a more stable contact angle over time. Interestingly, although four-layer coatings exhibited similar air permeability regardless of cationic group contents (Fig. 5), the contact angle results indicate that higher cationic group content leads to higher contact angles. This suggests that, despite extensive coverage of base paper micropores by CNFs after four coating layers resulting in comparable air permeability, CNFs coatings prepared with higher cationic group content provide improved surface smoothness and uniformity^46^. Gómez et al.^47^ also reported that reducing the surface roughness and efficiently smoothing the coating layer helped improve water resistance.

### Antibacterial performance of quaternized CNF-coated paper

**Fig. 7:**
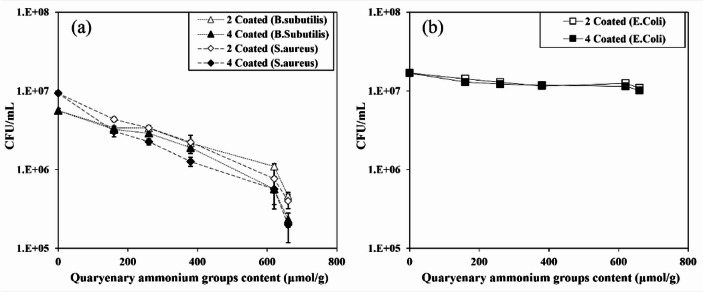
CFU counts on quaternized CNF-coated papers with different cationic group contents and coating layers. (Gram-positive: *B. subtilis* and *S. aureus* (**a**), Gram-negative: *E. coli* (**b**)).

Antibacterial tests were performed to evaluate the contact-active antibacterial performance of quaternized CNF-coated paper surfaces using *Escherichia coli* (Gram-negative) and *Bacillus subtilis* and *Staphylococcus aureus* (Gram-positive), which are widely used model strains for antibacterial evaluation. Figure 7 shows the results of CFU test according to the bacterial cell membrane structure. Figure 7a presents the antibacterial results for the Gram-positive strains (*B. subtilis* and *S. aureus*). For Gram-positive bacteria (Fig. 7a), antibacterial activity increased with both cationic group contents and coating layer number, indicating that antibacterial performance is strongly affected by cationic surface charge density and effective contact area provided by the coating structure. This trend agrees with the widely reported mechanism against cationic components. The antibacterial action of quaternary ammonium groups is generally attributed to a contact-active mechanism, in which positively charged ammonium groups are first attracted to negatively charged bacterial surfaces, followed by membrane insertion/disruption and leakage of intracellular constituents^30,49–51^. However, no clear dependence on cationic group content was observed for Gram-negative bacteria (Fig. 7b). This difference can be explained by the distinct cell envelope structures of Gram-positive and Gram-negative bacteria. Gram-positive bacteria possess a relatively porous peptidoglycan-rich envelope without an outer membrane, which allows easier access of contact-active cationic surfaces to the cytoplasmic membrane^49^. In contrast, Gram-negative bacteria are characterized by a thinner peptidoglycan layer and an additional outer membrane composed of lipoproteins, lipopolysaccharides, and phospholipids, which acts as an effective barrier against external substances, such as cationic components^52,53^.

**Fig. 8:**
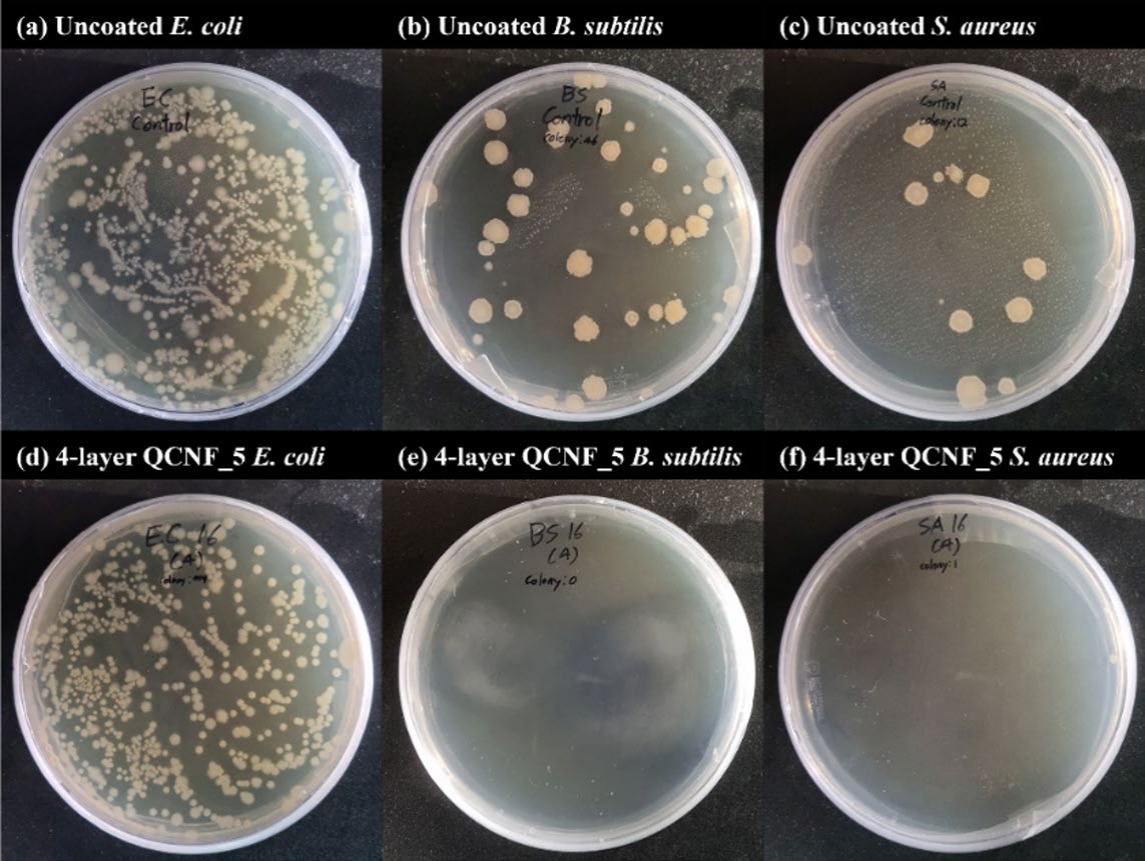
Antibacterial activity of quaternized CNFs-coated and uncoated papers against different bacterial strains (shaking flask assay).

In addition to the CFU counts test, a shaking flask assay was also performed to directly evaluate antibacterial activity under immersion conditions, where the coated paper was in continuous contact with bacterial suspensions. Figure 8 presents colony images obtained after bacterial suspensions were exposed to four-layer coated paper prepared using CNFs with the highest cationic group contents. Consistent with the CFU results, a clear antibacterial effect was observed against Gram-positive bacteria, while bacterial growth was evident in the case of Gram-negative bacteria. Therefore, in this study, although sufficient antibacterial activity against Gram-positive bacteria was achieved at a maximum cationic group content of approximately 660 µmol/g, a higher cationic group content may be required for effective antibacterial performance against Gram-negative bacteria.

## Conclusion

In this study, quaternized CNFs with different cationic group contents were successfully prepared and evaluated as coating materials for sustainable paper packaging applications. Increasing the cationic group content led to a reduction in cellulose DP, resulting in shorter fibrils and changes in suspension rheology, including decreased viscosity and yield stress. These rheological modifications facilitated improved flow behavior during coating, enabling the formation of smoother, more uniform, and continuous coating layers on paper substrates. As a result, the CNFs-coated papers exhibited enhanced barrier properties and improved water resistance. In addition, the quaternized CNFs-coated papers demonstrated strong contact-active antibacterial activity against Gram-positive bacteria, which increased with both cationic group contents and coating layer number. This behavior is likely due to the electrostatic interaction mechanism between cationic surfaces and negatively charged bacterial membranes. In contrast, antibacterial performance against Gram-negative bacteria remained limited, which is related to the presence of an outer membrane that hinders direct contact with cationic functionalities. These results indicate that higher cationic group contents or additional design strategies may be required to extend antibacterial performance toward Gram-negative bacteria.

## Data Availability

The datasets generated and/or analysed during the current study are not publicly available due to ongoing commercialization considerations requested by the collaborative research partner (Greenple), but are available from the corresponding author on reasonable request.
